# Identification of genomic loci associated with 21chlorophyll fluorescence phenotypes by genome-wide association analysis in soybean

**DOI:** 10.1186/s12870-018-1517-9

**Published:** 2018-11-29

**Authors:** Matthew Herritt, Arun Prabhu Dhanapal, Larry C. Purcell, Felix B. Fritschi

**Affiliations:** 1 Division of Plant Science, University of Missouri, Columbia, MO 65211 USA; 2 Department of Crop, Soil, and Environmental Sciences, University of Arkansas, Fayetteville, AR 72704 USA

**Keywords:** Chlorophyll fluorescence, Genome-wide association study, Single nucleotide polymorphisms, *Glycine max*

## Abstract

**Background:**

Photosynthesis is able to convert solar energy into chemical energy in the form of biomass, but the efficiency of photosynthetic solar energy conversion is low. Chlorophyll fluorescence measurements are rapid, non-destructive, and can provide a wealth of information about the efficiencies of the photosynthetic light reaction processes. Efforts aimed at assessing genetic variation and/or mapping of genetic loci associated with chlorophyll fluorescence phenotypes have been rather limited.

**Results:**

Evaluation of SoySNP50K iSelect SNP Beadchip data from the 189 genotypes phenotyped in this analysis identified 32,453 SNPs with a minor allele frequency (MAF) ≥ 5%. A total of 288 (non-unique) SNPs were significantly associated with one or more of the 21 chlorophyll fluorescence phenotypes. Of these, 155 were unique SNPs and 100 SNPs were only associated with a single fluorescence phenotype, while 28, 11, 2, and 14 SNPs, were associated with two, three, four and five or more fluorescence phenotypes, respectively. The 288 non-unique SNPs represent 155 unique SNPs that mark 53 loci. The 155 unique SNPs included 27 that were associated with three or more phenotypes, and thus were called multi-phenotype SNPs. These 27 multi-phenotype SNPs marked 13 multi-phenotype loci (MPL) identified by individual SNPs associated with multiple chlorophyll fluorescence phenotypes or by more than one SNP located within 0.5 MB of other multi-phenotype SNPs.

**Conclusion:**

A search in the genomic regions highlighted by these 13 MPL identified genes with annotations indicating involvement in photosynthetic light dependent reactions. These, as well as loci associated with only one or two chlorophyll fluorescence traits, should be useful to develop a better understanding of the genetic basis of photosynthetic light dependent reactions as a whole as well as of specific components of the electron transport chain in soybean. Accordingly, additional genetic and physiological analyses are necessary to determine the relevance and effectiveness of the identified loci for crop improvement efforts.

**Electronic supplementary material:**

The online version of this article (10.1186/s12870-018-1517-9) contains supplementary material, which is available to authorized users.

## Introduction

Photosynthesis is able to convert solar energy into chemical energy in the form of biomass, but the efficiency of photosynthetic total solar energy conversion is low. Zhu et al. [1], calculated theoretical maximum efficiencies of total solar radiation conversion into final biomass energy of 4.6 and 6.0% for C3 and C4 photosynthetic species, respectively. Most of the solar energy reaching earth’s surface is outside the spectrum of photosynthetically active radiation (PAR) and thus is not available for photosynthesis. Incident PAR can be absorbed, reflected or transmitted by plants, with the energy of most of the absorbed PAR available to drive photochemistry. As PAR increases, the percentage of absorbed quanta used for photosynthetic processes declines, resulting in dramatic differences in PAR use efficiency on diurnal and seasonal timescales [2]. Modeling photosynthesis of leaves of C3 species allowed calculations of the percentage of quanta used for carbon assimilation at various light levels. Under low levels of PAR, experienced by field-grown plants in the morning, 80% of the PAR is used for photosynthetic reactions while during mid-morning (when PAR is around 1000 μmol m^− 2^ s ^− 1^) only 25% is used for photosynthetic reactions. At mid-day (when PAR is around 2000 μmol m^− 2^ s ^− 1^) the efficiency can fall to or below 10% [3, 4], and excess light energy needs to be dissipated to avoid damage to the photosynthetic apparatus. Excess absorbed light energy can be dissipated through non-photochemical quenching (NPQ) mechanisms and to a lesser extent can be emitted as fluorescence [5].

Absorption of excess light can be damaging to plants through the generation of reactive oxygen species (ROS). Protective processes like NPQ reduce photosynthesis and lead to reduced carbon assimilation, but provide a safe route for excessive energy dissipation [6]. Losses in daily canopy carbon uptake resulting from reduced photosynthetic efficiency caused by NPQ response-dynamics to changes in light intensity that occur within a canopy were simulated to be between 12 and 30% [7]. Thus, increasing light use efficiency by reducing NPQ without heightening the incidence of photo-damage could have profound effects on crop productivity. Consequently, many researchers have explored ways to improve light use for biomass production through more advantageous NPQ or photoinhibition characteristics [8] and low temperature tolerance [9, 10]. Researchers have also sought to improve NPQ through metabolic engineering [11] and to improve recovery from NPQ through transgenic manipulation [12].

Aside from photochemistry and NPQ, light that is absorbed by chlorophyll can be re-emitted as fluorescence. This chlorophyll fluorescence can be measured using non-destructive techniques and has been investigated extensively to establish relationships with photosynthetic light dependent reactions [13–17]. While chlorophyll fluorescence and CO_2_ assimilation is well correlated under laboratory conditions, the relationship breaks down under field conditions [16, 18, 19]. Nonetheless, research to date indicates that, despite the relatively small loss of 1–2% of total absorbed light as chlorophyll fluorescence, important information about the light dependent reactions of photosynthesis can be gleaned from chlorophyll fluorescence measurements [20].

Chlorophyll fluorescence measurements can be used to track fluxes and efficiencies of processes from the initial absorption of light by chlorophyll molecules through the various steps of the electron transport chain [21]. Interrogations of light dependent reactions by chlorophyll fluorescence measurements are based on exposing dark-adapted photosynthetic samples to user-defined light intensities in a particular temporal pattern and quantification of the emission of light as chlorophyll molecules return from the excited to the relaxed state (fluorescence). The underlying molecular mechanisms of the light reaction processes and how they contribute to the rise in chlorophyll fluorescence are well understood [22]. For instance, after a photosynthetic tissue has been dark adapted, exposure to saturating light will induce a characteristic rise in chlorophyll fluorescence. This rise in fluorescence has been determined to be caused by the resulting reduction of electron acceptors within the photosynthetic light dependent reactions [22]. Two electron acceptors that have specifically been linked to rises in chlorophyll fluorescence over the one-second time scale are the primary quinone electron acceptor (Q_A_) that is located in the reaction center of photosystem II and plastoquinone, a mobile electron carrier that accepts electrons from photosystem II and donates them to cytochrome b_6_f [20]. Once these electron acceptors are reduced, they are not able to accept another electron. As photons continue to be absorbed by chlorophyll molecules and more electrons enter the electron transport chain, the pools of electron acceptors become fully reduced, electrons back up in upstream steps, and eventually impede the transfer of absorbed light energy to the reaction center of photosystem II, in turn resulting in more re-emission of the absorbed light as fluorescence. Ultimately, high chlorophyll fluorescence represents a smaller portion of the absorbed quanta being used for photochemistry [20].

The relative ease of chlorophyll fluorescence measurements fosters widespread adoption for quantitative analyses of photosynthetic light dependent reactions. Indeed, chlorophyll fluorescence measurements are now widely used to study plant responses to a broad range of environmental conditions, including heat stress [23], cold stress [24], drought stress [25, 26] and nitrogen deficiency [27]. In contrast to the substantial body of work on the impact of plant stress on chlorophyll fluorescence, a comprehensive analysis of the genetic factors underlying chlorophyll fluorescence characteristics of field grown crop species is lacking. Nevertheless, a number of studies have reported genetic markers associated with select chlorophyll fluorescence phenotypes. For wheat (*Triticum aestivum*) grown under controlled environment conditions, Azam et al. [28]identified 13 quantitative trait loci (QTL) for four chlorophyll fluorescence phenotypes in seedlings of a biparental population characterized at 25 °C, and an additional 24 QTL when heat stressed (38 °C). Šimić et al. [29] characterized a maize (*Zea mays*) mapping population of 205 recombinant inbred lines grown in four field environments and identified 10 QTL for seven chlorophyll fluorescence phenotypes. For soybean, two research groups have reported genetic markers associated with selected chlorophyll fluorescence phenotypes. Yin et al. [30] phenotyped a biparental mapping population and found 26 QTL for ten chlorophyll fluorescence phenotypes, and Hao et al. [31] identified 51 SNPs for five chlorophyll fluorescence phenotypes in soybean using genome-wide association study (GWAS). Neither of these soybean studies examined a comprehensive set of chlorophyll fluorescence phenotypes, and both of them characterized fluorescence during late reproductive development (R6, full seed) when green leaf area index and daily gross primary production have already started to decline [32, 33].

The involvement of chloroplast encoded gene products in photosynthesis limits the power of GWAS to elucidate the genetics underlying chlorophyll fluorescence and complicates crop improvement efforts that target photosynthesis traits [34]. That is, chloroplastic genes that contribute to variation in chlorophyll fluorescence phenotypes would not be detected through genetic studies of the nuclear genome. Among the approximately 100 chloroplastic genes are 28 genes encoding thylakoid proteins and the large rubisco subunit [34]. Nonetheless, nuclear genes encode the vast majority of proteins found in the chloroplast [35], and therefore, these genes can be captured with GWAS and may be leveraged for breeding efforts targeting nuclear-encoded genes.

This study was conducted to identify genomic regions associated with photosynthetic light dependent reactions at a developmental stage (beginning to full bloom; R1-R2 stage) when soybean leaf photosynthetic rates are at their peak. To this end, a panel of diverse soybean genotypes was grown in four different environments and 21 chlorophyll fluorescence phenotypes were measured and mapped by genome-wide association (GWA) analysis. The genetic markers and genotypes with advantageous photosynthetic light reaction characteristics identified in this study can serve as a resource for those researching soybean photosynthesis, and for improving soybean performance on the basis of photosynthetic characteristics.

## Materials & methods

### Locations and experimental design

Field experiments were conducted at three locations in Missouri, USA and one location in Arkansas, USA in 2013. In Missouri, experiments were conducted at Rollins Bottom (Rollins) in Columbia (38°55′37.5”N 92°20′44.6”W) on a Haymond silt loam soil (course-silty, mixed, superactive, mesic Dystric Fluventic Eutrudepts), at Rhodes Farm (Rhodes) near Clarkton (36°48′78.7” N, 89°96′32.8” W) on a Malden fine sand (Mixed, thermic Typic Udipsamments) and at the Bradford Research Centre (Bradford) near Columbia (38°89′44.2” N 92°20′54.7” W) on a Mexico silt loam soil (fine, smectitic, mesic Vertic Epiaqualfs). In Arkansas, soybean were grown near Stuttgart at the Rice Research and Extension Center (Stuttgart) (34°47′52.7” N, 91°41′81.7” W) on a Crowley silt loam soil (fine, smectitic, thermic Typic Albaqualfs). Weather data for Bradford, Rollins, and Rhodes were accessed from weather archives at http://agebb.missouri.edu/weather/stations/ from the Columbia – Bradford Farm, Columbia – Sanborn Field, and Clarkton stations that were located within 1 km, 3 km, and 1 km of the fields, respectively [36]. Since data for daily solar radiation were not available for the Columbia – Bradford Farm station, daily solar radiation data for the Bradford location were obtained from the Columbia – Jefferson Farm station located within 5.5 km of the field at Bradford. Weather data from a station located within 2 km of the field at the Stuttgart location were obtained from archives of the United States Department of Agriculture (USDA) Agricultural Research Service via https://www.ars.usda.gov/southeast-area/stuttgart-ar/dale-bumpers-national-rice-research-center/docs/weather-stations/ [37].

The seeds of 189 maturity group (MG) IV soybean accessions, originally obtained from the USDA Soybean Germplasm Collection, were sown approximately 2.5 cm deep at a density of 25 seeds m^− 2^ in rows 0.76-m apart in tilled fields at all locations. At Bradford and Rollins, genotypes were planted in four row plots measuring 6.1 m and 2.4 m in length, respectively. At Rhodes and Stuttgart plots consisted of single rows that were 2.1 m and 4.6 m long, respectively. The accessions were planted in a randomized complete block design with three replications at Rollins, Rhodes, and Stuttgart, and one replication at Bradford on 11 June 2013, 23 May 2013, 31 May 2013, and 8 June 2013, respectively. Fertilizer applications were based on soil test-based recommendations of the University of Missouri (Rollins, Rhodes, Bradford; http://aes.missouri.edu/pfcs/soiltest.pdf) and the University of Arkansas (Stuttgart; http://www.uaex.edu/publications/pdf/mp197/chapter5.pdf) and did not include any applications of N. Weeds were controlled using pre- and post-emergence herbicide applications as previously described [38]. Experiments at Rollins, Rhodes, and Bradford were rainfed while the field at Stuttgart was furrow irrigated to maintain well-watered conditions. A summary of weather data from the locations can be found in Table 1 from planting till the last day of fluorescence measurements and a summary of weather data three days prior to beginning of fluorescence measurements.

**Table 1:** Summary of environmental conditions for the growing season and three days prior to fluorescence measurements for the four environments

Environment	Cumulative Precipitation	Max air temperature	Min air temperature	Average total daily solar radiation
	mm	°C	°C	MJ m^−2^
Planting through fluorescence measurements				
Bradford	134	30.0	17.8	20.5
Rhodes	163	30.9	18.8	21.3
Rollins	137	30.8	17.2	20.0
Stuttgart	57	31.2	21.2	24.2
Three days prior to fluorescence measurements				
Bradford	3	27.3	19.3	14.8
Rhodes	12	31.3	21.4	16.0
Rollins	5	29.6	15.5	18.3
Stuttgart	0	31.2	21.3	21.3

Precipitation, maximum air temperature, minimum air temperature, and total daily solar radiation data were obtained from the nearest weather stations with available data

### Genotypes and chlorophyll fluorescence measurements

Chlorophyll fluorescence phenotypes of 189 soybean genotypes were collected. The genotypes originated from 10 different countries including 97 from South Korea, 42 from China, 28 from Japan, 9 from North Korea, six from Georgia, two each from Russia and Taiwan, and one each from India, Mexico, and Romania. The 189 soybean genotypes were a subset of 373 genotypes that were selected based on GRIN (Germplasm Resources Information Network, www.ars-grin.gov) data with genotypes falling either into a group that included genotypes with good seed yield and agronomic characteristics, or a group that included genotypes selected considering geographical origin without consideration of yield but while maintaining good agronomic characteristics such as height, lodging, and shattering [38]. All these genotypes have previously been genotyped using a 50 K SNP chip as described in Song et al. [39], and these SoySNP50K data (available at: https://soybase.org/snps/download.php) were used for analyses as described below. The 189 genotypes used for chlorophyll fluorescence measurements were selected following analysis using the SoySNP50K dataset (results not shown) to represents much of the genetic diversity within the 373 genotypes.

Chlorophyll fluorescence measurements were made on clear, sunny days 63 and 64 days after planting (DAP) at Rhodes, 63, 64, and 65 DAP at Stuttgart, 64 and 65 DAP at Bradford, and 64, 65 and 66 DAP at Rollins when genotypes were at beginning bloom to full bloom (R1 to R2) [40]. Two measurements were made with Fluorpen Z995-PAR (Qubit systems INC, Kinston Ontario, Canada) fluorometers in each plot on center leaflets of the uppermost fully expanded leaves following 20 min of dark adaptation imposed with detachable leaf clips. All measurements were made on sunny days between 10:00 and 14:00 Central Standard Time (CST).

### Statistical analysis

Statistical analyses were conducted using SAS 9.4 (SAS institute Inc. 2004). PROC MEANS was used to generate basic descriptive statistics for the chlorophyll fluorescence phenotypes based on the average of each genotype for each location. Analysis of variance was performed for genotype, environment and genotype x environment using PROC GLM. Best linear unbiased predictions (BLUPs) were used for GWAS. To this end, PROC GLIMMIX with location as the fixed effect and all other factors as random was used to generate across-location BLUPs. Broad sense heritability estimates (H^2^) for chlorophyll fluorescence phenotypes were calculated according to Piepho and Mohring [41].

### Population structure

STRUCTURE, a Bayesian model-based software program, was used to infer the population structure of 189 genotypes using 32,453 SNPs [42]. The population structure analysis was performed using an admixture and allele frequency correlated model with 100,000 burn-in iteration and Markov chain Monte Carlo (MCMC) method using five independent iterations with the hypothetical number of subpopulations (k) ranging from 1 to 10. The soybean genotypes were assigned to a subpopulation based on k = 7, obtained from the rate of change of log probability data [LnP(D)] between successive k values at which LnP(D) reached a plateau. Based on the optimum k (k = 7), the population structure matrix (Q) was generated for further genome-wide association analyses. TASSEL 5 software [43, 44] was used to generate the kinship matrix based on scaled identity-by-state similarity matrix as described [45].

### Genome-wide association analysis

The GWA analysis was performed using the R-package GAPIT (Genome Association and Prediction Integrated Tool) [43, 46]. The co-variate Q was obtained from the STRUCTURE run, and the kindship matrix (K) was calculated using the VanRaden method to determine the relatedness among individuals. A compressed mixed linear model (CMLM) incorporating the kinship matrix (K) to model random effects and the population structure (Q) to model fixed effects, was used for GWA analysis [42, 43, 46, 47]. Multiple testing was conducted to assess the significance of marker phenotype associations using QVALUE R 3.1.0 (http://genomics.princeton.edu/storeylab/qvalue/windows.html) employing the smoother method [48], an extension of the FDR method [49]. Single nucleotide polymorphisms with an FDR < 0.05 were considered significant and all markers that satisfied the multiple testing had –log10 *P* values ≥3.10.

### Candidate genes

To identify genes that may be affecting the chlorophyll fluorescence phenotypes, a region encompassing ±1 Mb from each of the multi-phenotype loci was searched for genes annotated with relation to photosynthesis, light dependent reactions, starch or sugar metabolism and chlorophyll metabolism in SoyBase (www.soybase.org) [50].

## Results

### Phenotypes and heritability

**Fig. 1:**
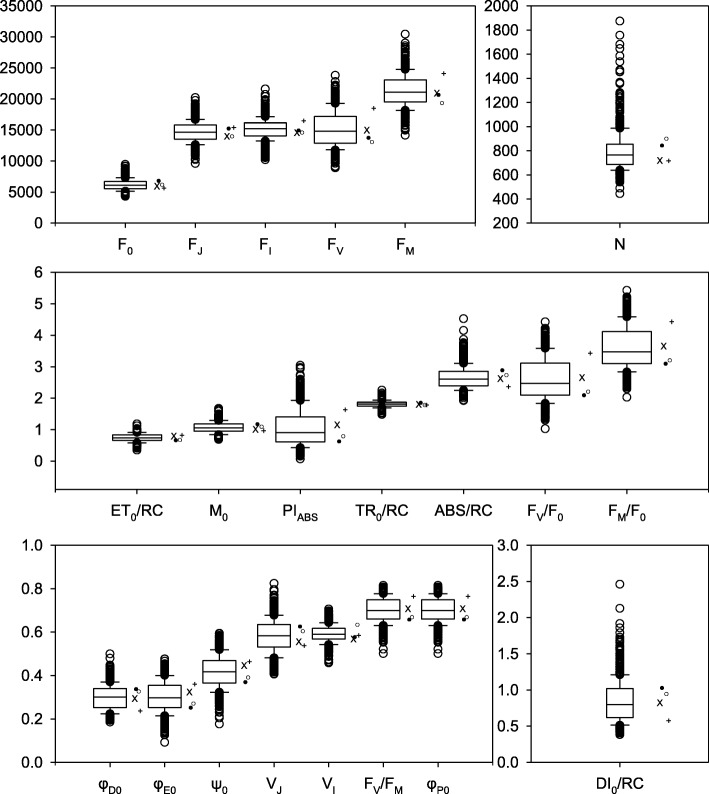
Box and whisker plots for all chlorophyll fluorescence phenotypes across all four environments. Lines within boxes are the mean across all four environments. The top and bottom of the boxes represent the 1st and 3rd quartile and whiskers represent 1.5 times the difference between 3rd and 1st quartile. Open circles indicate outlier data beyond the whiskers. The means for Bradford, Rhodes, Rollins and Stuttgart locations are represented by +, X, •, and ⚬ respectively. To simplify the interpretation of the data relative fluorescence was excluded from the Y axis of the uppermost graph as all other phenotypes are unitless

A total of 21 chlorophyll fluorescence phenotypes were measured for 189 genotypes in four different environments in 2013. A brief description with calculations for each chlorophyll fluorescence phenotype is provided in Table 2. Significant genotype and environment effects were observed for all phenotypes and only for TR_0_/RC was the interaction not significant. Despite significant environmental effects for all of the phenotypes, the range relative to the mean for the different phenotypes extended from a low of 13% for V_I_ to 112% for PI_ABS_ (Fig. 1). Among the primary fluorescence phenotypes (F_0_, F_J_, F_I_, and F_M_) F_0_ had the highest range relative to the mean with 47% and F_M_ had the lowest with 26%. The Relative fluorescence phenotypes had ranges relative to their means of 37, 52 and 20% for F_M_/F_0_, F_V_/F_0_ and F_V_/F_M_, respectively (Fig. 1). The phenotypic range of the Extracted phenotypes Mo, N, φ_Do_, φ_Eo_, Ψ_0_, and PI_ABS_, were all greater than 43% relative to the mean, except for φ_Po_ (20%). The Energy flux phenotypes ABS/RC, TR_0_/RC, ET_0_/RC, and DI_0_/RC exhibited diverse ranges relative to their mean values of 41, 18, 29 and 93%, respectively.

**Table 2:** Calculations and definitions of fluorescence phenotypes (Strasser et al. 2000) with categorization, broad sense heritability (H^2^) and *p*-values of genotype (G), environment (E) and genotype by environment interaction (GxE) effects on fluorescence phenotypes

	Phenotype category	Phenotype	Calculation	Definition	H^2^ (%)	G	E	GxE
1	Primary fluorescence phenotypes	F_0_		Minimum Fluorescence	36.8	<.0001	<.0001	<.0001
2		F_J_		Fluorescence intensity at the J-step (2 ms)	29.5	<.0001	<.0001	<.0001
3		F_I_		Fluorescence intensity at the I-step (60 ms)	22.8	<.0001	<.0001	<.0001
4		F_M_		Maximum fluorescence	20.6	<.0001	<.0001	<.0001
5		F_V_	*F*_*M*_ − *F*_0_	Variable fluorescence	17.3	<.0001	<.0001	<.0001
6		V_J_	(*F*_*J*_ − *F*_0_)/(*F*_*M*_ − *F*_0_)	Variable Fluorescence at the J-step	16.3	<.0001	<.0001	<.0001
7		V_I_	(*F*_*I*_ − *F*_0_)/(*F*_*M*_ − *F*_0_)	Variable fluorescence at the I-step	14.6	<.0001	<.0001	<.0001
8	Relative phenotypes	F_M_/F_0_			20.7	<.0001	<.0001	<.0001
9		F_V_/F_0_	(*F*_*M*_ − *F*_0_)/*F*_0_	Maximum efficiency of photochemistry	20.8	<.0001	<.0001	<.0001
10		F_V_/F_M_	(*F*_*M*_ − *F*_0_)/*F*_*M*_	Maximum yield of primary photochemistry	23.3	<.0001	<.0001	<.0001
11	Extracted phenotypes	M_0_	4(*F*_300_*μs* − *F*_0_)/(*F*_*M*_ − *F*_0_)	Rate of reaction center closure	23.4	<.0001	<.0001	<.0001
12		N	(*Area*/(*F*_*M*_ − *F*_0_))*x M*_0_ *x*(1/*V*_*J*_)	turn-over number Q_A_ reduction events between time 0 and Fm	2.4	0.0002	<.0001	0.0194
13		φ_Po_	1 − (*F*_0_/*F*_*M*_)	Maximum yield of primary photochemistry (Fv/Fm)	4.6	<.0001	<.0001	<.0001
14		ψ_0_	1 − *V*_*J*_	Likelihood that a trapped exciton can move an electron further than Q_A_^−^	2.5	<.0001	<.0001	<.0001
15		φ_Eo_	(1 − (*F*_0_/*F*_*M*_))*x Ψ*_0_	Quantum yield of electron transport	2.6	<.0001	<.0001	<.0001
16		φ_Do_	1 − *φ*_*Po*_	Quantum yield at time 0 for energy dissipation	4.6	<.0001	<.0001	<.0001
17		PI_ABS_	$\left(\frac{RC}{ABS}\right)x\left(\frac{\varphi_{Po}}{1-{\varphi}_{Po}}\right)x\left(\frac{\varPsi_0}{1-{\varPsi}_0}\right)$	Performance Index of PSII normalized for equal absorption	3.2	<.0001	<.0001	<.0001
18	Energy flux phenotypes	ABS/RC	*M*_0_*x*(1/*V*_*J*_)*x*(1/*φ*_*Po*_)	Energy absorption by antenna per reaction center	6.2	<.0001	<.0001	<.0001
19		TR_0_/RC	*M*_0_*x*(1/*V*_*J*_)	Flux of excitons trapped per reaction center	4.7	<.0001	<.0001	0.1487
20		ET_0_/RC	*M*_0_*x*(1/*V*_*J*_)*x Ψ*_0_	Energy flux for electron transport per reaction center	0.4	<.0001	<.0001	<.0001
21		DI_0_/RC	(*ABS*/*RC*) − (*TR*_*o*_/*RC*)	Flux ratio of energy dissipation per reaction center	4.7	<.0001	<.0001	<.0001

The Broad sense heritability estimates (H^2^) based on combined data from all four environments varied between less than 1% for ET_0_/RC and 36.8% for F_0_ (Table 2). Across all of the fluorescence phenotypes reported in this study the average H^2^ was 13.4%.

### Fluorescence phenotype correlations

**Fig. 2:**
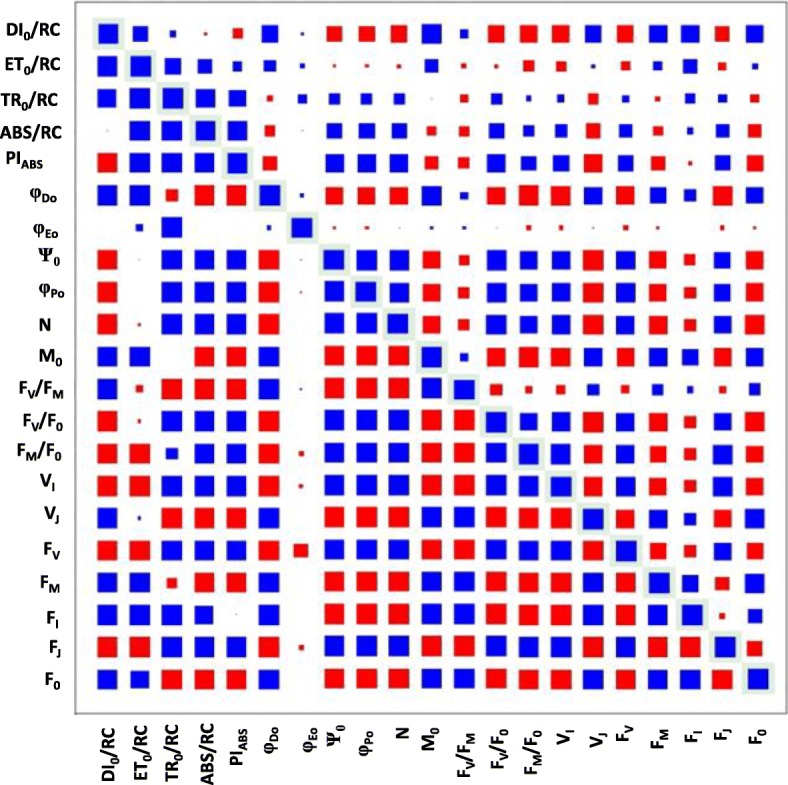
Correlation coefficient and p-values for all chlorophyll fluorescence phenotypes. Outlined boxes indicate the box size representing a 1.0 correlation coefficient or a p-value below 0.05. Boxes above the correlation diagonal visualize the correlation coefficients for each respective phenotype combination. Boxes below the correlation diagonal indicate *p*-values. A blue box indicates a positive relationship and a red box indicates a negative relationship. The size of the box is proportional to the strength of the relationship for both correlation coefficients and *p*-values

All 21 chlorophyll fluorescence phenotypes determined provide information about the light reaction processes of photosynthesis, and 14 of them are ratios or otherwise calculated based on primary phenotypes (Table 2). As such, these phenotypes are not independent of each other and many significant correlations can be expected. To examine the level of connection between phenotypes, pairwise correlations of all the fluorescence phenotypes were computed, providing perspective relative to the results of the association analyses, in particular regarding SNPs that were associated with multiple phenotypes. As illustrated in Fig. 2, most of the phenotypes were strongly correlated with at least some of the other phenotypes. For 11 of the fluorescence phenotypes, r values were > 0.75 for correlations with more than 75% of the other phenotypes. Two phenotypes, Ψ_0_ which represents the quantum yield of electron transport flux from Q_A_ to Q_B_, and V_J_ which represents the variable fluorescence at the J step, were highly correlated (*r* > 0.75) with 80% of the other phenotypes. In contrast, the number of turnover events of Q_A_ (N) and the variable fluorescence at the I step (V_I_) were not highly correlated with any of the other fluorescence phenotypes.

### Population structure analysis

**Fig. 3:**
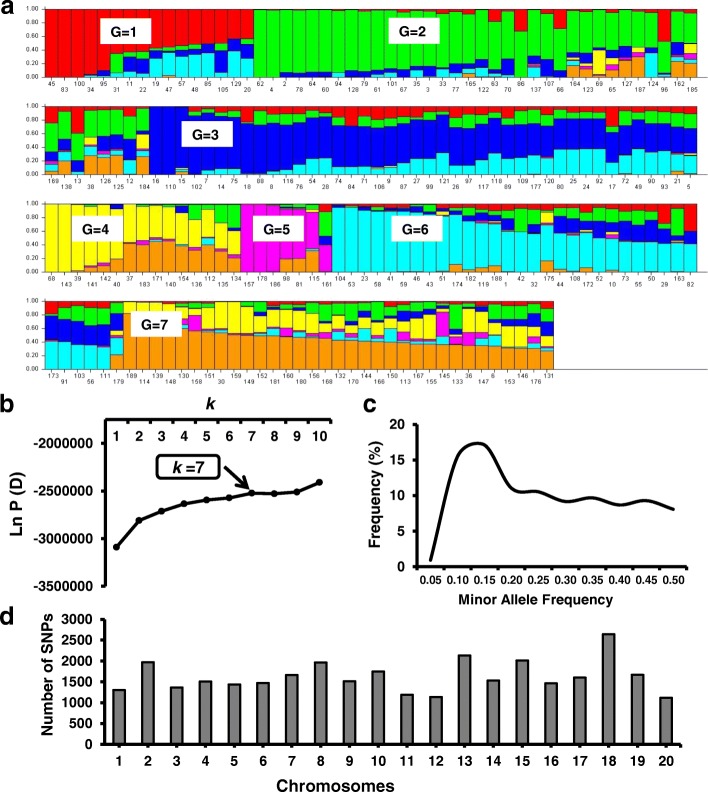
Population structure results using 32,453 SNPs across 189 soybean genotypes. **a** Model-based clustering method STRUCTURE showing individual genotypes in the population structure of 189 soybean genotypes (*k* = 7) based on 32,453 single nucleotide polymorphisms. The *y*-axis is the subpopulation membership, and the *x*-axis is the individual genotypes in each subpopulation. G, subpopulation (G1–G7). **b** Log probability data LnP(D) as function of k (number of groups) from the structure run. The plateau of the graph at k = 7 indicates the minimum number of subgroups possible in the panel. **c** Minor Allele Frequency percentage distribution of 32,453 SNPs. **d** Distribution of 32,453 SNPs obtained from 189 soybean genotypes across 20 soybean chromosomes

The analysis to estimate the most likely number of subpopulations among the 189 genotypes was conducted with STRUCTURE software using 32,453 SNPs distributed across the 20 soybean chromosomes. The number of subpopulations was determined by plotting the estimated likelihood value [LnP(D)] obtained from STRUCTURE runs against *k*, indicating that *k = 7* was optimal (Fig. 3). The estimated population structure revealed genotypes with membership to multiple subpopulations and few subpopulations exhibiting distinctive identities (Fig. 3a, Table 3). Among the different subpopulation groups (G), G2 and G3 contained the largest number of genotypes (42 in each) and G5 encompassed the smallest number of genotypes (7). One subpopulation (G1) included individuals exclusively from South Korea while all other groups comprised genotypes from multiple countries of origin (Table 3, Fig. 3a).

**Table 3:** Countries of origin for the 189 genotypes arranged by sub-population group as determined by the STRUCTURE analysis using 32,453 SNPs

Sub-Population Groups	Number of Individuals	Country of Origin Distribution
G1	16	16 South Korea (100%)
G2	42	2 China (4.76%); 2 Georgia (4.76%); 25 Japan (59.52%); 2 North Korea (4.76%); 1 Russia (2.38%); 9 South Korea (21.42%); 1 Taiwan (4.76%)
G3	42	1 China (2.38%); 1 India (2.38%); 1 Romania (2.38%); 39 South Korea (92.86%)
G4	15	9 China (60%); 3 Georgia (20%); 1 Japan (6.66%); 1 Russia (6.66%); 1 South Korea (6.66%)
G5	7	2 China (28.57%); 1 North Korea (14.28%); 1 Mexico (14.28%); 3 South Korea (42.85%)
G6	34	2 China (5.88%); 1 Japan (2.94%); 31 South Korea (91.17%)
G7	33	26 China (78.78%); 1 Georgia (3.03%); 1 Japan (3.03%); 1 North Korea (3.03%); 3 South Korea (11.53%); 1 Taiwan (3.03%)

### Genome-wide association analysis

**Fig. 4:**
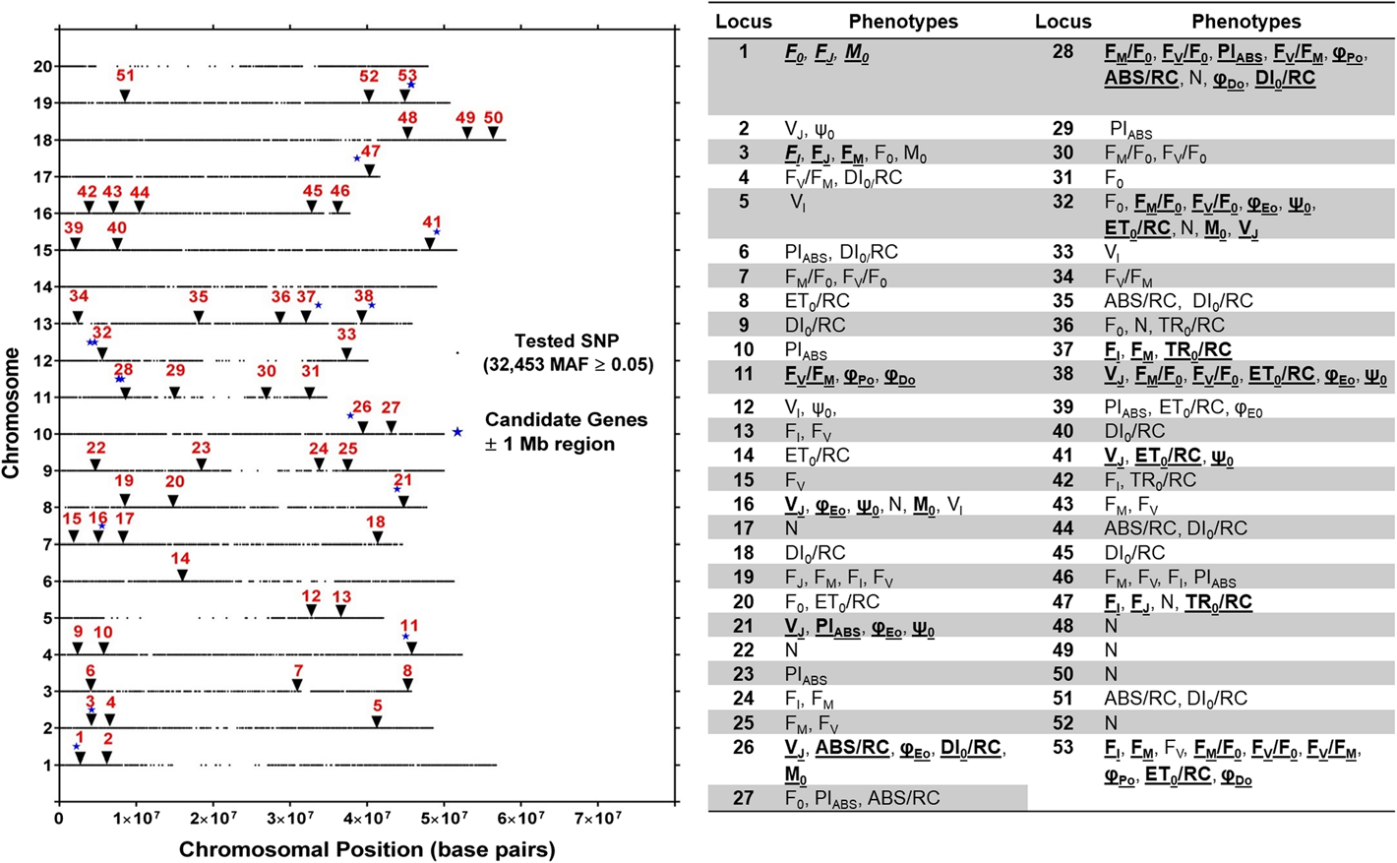
Location of putative loci significantly associated with one or more of 21 chlorophyll fluorescence phenotypes and 30 chlorophyll fluorescence related genes identified in Soybase and the KEGG pathway of chlorophyll biosynthesis. Loci are indicated by numbers from 1 to 53 from left to right positioned above the respective chromosomes starting with Chromosome 1. For each chromosome, the black dots represent the locations of SNPs evaluated for association with chlorophyll fluorescence. The phenotypes within each locus are also listed. Loci with bold and underlined phenotypes indicate phenotypes significantly associated with same SNP identified for at least three phenotypes within loci

Evaluation of SoySNP50K iSelect SNP Beadchip data from the 189 genotypes phenotyped in this study identified 32,453 SNPs with a minor allele frequency (MAF) ≥ 5%. The number of genotypes with MAF was relatively stable in the MAF range of 0.15 to 0.50 (Fig. 3c). The number of SNPs per chromosome averaged about 1600 and was highest for chromosome 18 (2643) and lowest for chromosome 20 (1116) (Fig. 3d). The distribution of SNPs within each chromosome is evident from Fig. 4, revealing lower SNP densities in centromeric regions.

The 32,453 SNPs with MAF ≥ 5% were used for marker-phenotype association analyses with the BLUP mean across all four environments. A compressed mixed linear model (CMLM) accounting for population structure (Q-matrix) and genetic relatedness (K-matrix) was applied to help avoid false positives. The markers that showed a significant (−log10 *p* value > 3.10) association were considered putative marker-phenotype associations (MPAs).

A total of 288 significant SNP-trait associations were identified for the 21 chlorophyll fluorescence phenotypes (Table 4). Of these, 100 SNPs were only associated with a single fluorescence phenotype, while 28, 11, 2, and 14 SNPs, were associated with two, three, four and five or more fluorescence phenotypes, respectively. Among all 21 chlorophyll fluorescence phenotypes, the number of turnover events (N) was associated with the largest number of SNPs (39 SNPs) and the variable fluorescence at the I step (V_I_) with the lowest number of SNPs (3 SNPs) (Table 4). Significant associations were identified on every chromosome, except for Chromosomes 14 and 20. With 41 SNPs, the greatest number of associations were found on Chromosome 19 and the lowest number of associations were found on Chromosome 6 (1 SNP) (Table 5).

**Table 4:** Summary of phenotype significant SNPs association and location of chromosome harbouring significant SNPs

Phenotype	Number of SNPs with significant association	Number of Chromosomes harbouring significant SNPs
ABS/RC	9	5
DI_0_/RC	16	9
ET_0_/RC	15	7
F_I_	20	8
F_J_	5	4
F_M_	18	6
F_M_/F_0_	12	5
F_0_	9	7
F_V_	25	6
F_V_/F_M_	13	5
F_V_/F_0_	12	5
M_0_	12	5
N	39	8
φ_Do_	9	3
φ_Eo_	12	6
φ_Po_	9	3
PI_ABS_	14	8
Ψ_o_	13	7
TR_0_/RC	8	3
V_I_	3	3
V_J_	15	8

**Table 5:** Distribution of unique SNPs association on 20 soybean chromosomes and number of phenotypes observed for Unique SNPs

Soybean Chromosome Numbers	Number of Unique SNPs with significant association	Number of Phenotypes for Unique SNPs identified
Gm01	2	5
Gm02	6	8
Gm03	3	4
Gm04	4	5
Gm05	3	4
Gm06	1	1
Gm07	6	7
Gm08	13	11
Gm09	7	5
Gm10	4	7
Gm11	9	10
Gm12	16	10
Gm13	9	14
Gm14	0	0
Gm15	7	6
Gm16	13	7
Gm17	9	4
Gm18	3	1
Gm19	41	12
Gm20	0	0

The 288 non-unique SNPs represent a total of 155 unique SNPs contributing to 53 loci, 14 of which were identified based on single SNPs and the remaining 39 by at least two and up to 22 SNPs. Of the 53 loci, 15 were associated with a single fluorescence phenotype, 19 with two fluorescence phenotypes, and another 19 with three or more phenotypes (Additional file 1 and Fig. 4). The 155 unique SNPs included 27 that were associated with three or more phenotypes, and thus were called multi-phenotype SNPs (Table 5I). These 27 multi-phenotype SNPs marked 13 multi-phenotype loci (MPL) identified by individual SNPs associated with multiple chlorophyll fluorescence phenotypes or by more than one SNP located within 0.5 MB of other multi-phenotype SNPs (Additional file 2).

Multi-phenotype locus 13 identified on chromosome 19 was associated with nine phenotypes. Across the six multi-phenotype SNPs associated with MPL-13, all shared Fv. The MPL-7 on chromosome 11 was comprised of five SNPs associated with multiple phenotypes, and all of the SNPs shared F_V_/F_M_ and at least four and up to six other phenotypes associated with this locus. Chromosome 13 was the only chromosome that had two MPL, namely MPL-9 and MPL-10, which were associated with three and six phenotypes, respectively.

### Candidate genes

A search for genes that are associated with photosynthesis and may modulate chlorophyll fluorescence was conducted for the 13 loci associated with three or more fluorescence phenotypes. This search identified 30 candidate genes that were located within ±1 Mb of the 13 MPL (Table 7). The annotations indicated that eight genes were related to chlorophyll biosynthesis, seven genes encode photosystem II proteins, and three genes are involved with chloroplast structure or development. The remaining genes included some that were annotated as Fructose-bisphosphate aldolase 2, Sucrose synthase 3, large subunit of Rubisco, Cytochrome b6f, a protein involved in the assembly of chloroplast ATP synthase, and genes that are involved in the regulation of photosynthesis.

## Discussion

Photosynthetic light dependent reactions are sensitive to changes in environmental conditions and chlorophyll fluorescence measurements have been used extensively to examine the influence of a broad range of stress factors on the light dependent reactions [20]. Indeed, the impact of abiotic stress factors such as low [24] and high temperatures [23], salt [51], drought [25, 26] and nutrient deficiency [27] on plant growth and productivity can be mediated, at least in part, through effects on photosynthetic light dependent reactions. The substantial body of work that has linked plant productivity under adverse conditions with detrimental impacts on photosynthetic light dependent reactions, suggests that chlorophyll fluorescence measurements may provide information useful for selection of more stress tolerant genotypes in breeding programs [30, 31, 52–54]. However, despite this widespread use of chlorophyll fluorescence measurements and the ability to rapidly phenotype a large number of plants, efforts aimed at assessing genetic variation and/or mapping of genetic loci associated with chlorophyll fluorescence phenotypes have been rather limited. Nonetheless, QTL for photosynthetic light reaction phenotypes have been mapped successfully for a few crop species, including wheat [28, 54–57], maize [29], sunflower [58] and soybean [30, 31]. Heritability estimates provided by these studies generally range between 10 and 90%, and indicate that selection and breeding for chlorophyll fluorescence traits is possible.

### Fluorescence phenotypes and heritability

Knowledge regarding natural genotypic variation in chlorophyll fluorescence phenotypes in soybean is very limited. In the present study, 21 chlorophyll fluorescence phenotypes were assessed in a diverse panel of 189 soybean genotypes grown in four different environments. Significant genotype and environment effects were observed for all 21 phenotypes and significant genotype by environment interactions for all but TR_o_/RC (Table 2). Previous studies by Yin et al. [30] and Hao et al. [32] assayed soybean plants during late reproductive growth and were limited to ten and five chlorophyll fluorescence phenotypes, respectively. The only chlorophyll fluorescence phenotypes in common between these two and the present study were F_V_/F_M_, ABS/RC, and PI_ABS_. With 0.14 and 1.10, the phenotypic ranges observed in this study for F_V_/F_M_ and ABS/RC, respectively, exceeded the ranges of 0.09 (F_V_/F_M_) and 0.989 (ABS/RC) reported by Hao et al. [32] and 0.08 (F_V_/F_M_) and 0.91 (ABS/RC) observed by Yin et al. [30]. The range in PI_ABS_ observed in this study, 1.12, was narrower than the ranges observed in both previous studies (5.41 by Hao et al. [31]; 5.05 by Yin et al. [30]. In a QTL mapping study involving 150 double haploid lines of wheat under well-watered conditions, [54] found a range of F_V_/F_M_ of 0.14 which is identical to the present study. The phenotypic ranges documented here were comparable to or exceeded those reported by several research groups for other species, including for F_V_/F_0_ for which a range of 1.32 was found in the present study compared to a range of 0.20 observed among four greenhouse-grown barley (*Hordeum vulgare* L.) genotypes that had been selected for differing grain yield under drought stress [59]. With 5583 (F_V_) and 0.14 (F_V_/F_M_), the ranges documented in the present study also exceed those of four potato genotypes (ranges of 451 for F_V_ and 0.11 for F_V_/F_M_) that differed in response to nitrogen levels [60].

In general, broad sense heritability values for chlorophyll fluorescence phenotypes vary considerably [61–63]. In the present study, heritabilities were determined to range between 0.4% (ET_0_/RC) and 36.8% (F_0_). Hao et al. [32], reported broad sense heritabilities ranging from 37.6 to 41.1% for five chlorophyll fluorescence traits in soybean, exceeding the heritabilities found in the present study. However, it is important to note that Hao et al. [32] assayed chlorophyll fluorescence relatively late in development (R6; full seed) which may partly explain the greater heritabilities but may also have confounded the genetics underlying photosynthesis with senescence related loci. The significant natural variation in each chlorophyll fluorescence phenotype observed among the diverse soybean genotypes, coupled with broad sense heritabilities of the primary chlorophyll fluorescence phenotypes and the albeit low heritabilities of the Relative, Extracted and Flux phenotypes, indicate that selection and breeding for these traits should be possible.

### Chlorophyll fluorescence SNPs and loci

Genome-wide association analysis identified 288 SNPs that were associated with at least one of 21 chlorophyll fluorescence phenotypes. Marker-trait associations were absent on chromosomes 14 and 20 but were found on all other chromosomes. The 288 total SNPs identified across all four environments comprised 153 unique SNPs, which marked 53 loci. Fourteen of the 53 loci were identified based on single SNPs and 39 by at least two and up to 22 SNPs. Of the 53 loci, 19, 9, and 25 were associated with a single, two, and three or more phenotypes, respectively (Fig. 4; Additional file 1). Twenty-seven of the 153 total unique SNPs were associated with three or more phenotypes. These 27 multi-phenotype SNPs represented 13 MPL (Additional file 2). Since the chlorophyll fluorescence phenotypes all provide information about photosynthetic light dependent reactions, significant correlations between many of the fluorescence phenotypes are not surprising (Fig. 2) and consistent with numerous SNPs that were common among chlorophyll fluorescence phenotypes. These MPL could be marking individual genes that affect all of the associated chlorophyll fluorescence phenotypes or the MPL could indicate the presence of multiple genes that affect the phenotypes [64]. The MPL identified here, regardless whether they mark one or multiple genes, should be useful to improve soybean photosynthetic efficiency.

Previous studies have shown that highly correlated phenotypes often have genetic markers that group together [30, 53, 54, 65]. Similar to Hao et al. [32], a number of significant SNPs identified in the present GWAS were associated with three or more fluorescence phenotypes, and thus were identified as 13 MPL (Table 6). Because they were identified by multiple phenotypes, these MPL were considered particularly promising. Six of these 13 MPL were associated with three phenotypes, two loci with four phenotypes, and one locus each with five, six, seven, eight and nine phenotypes.

**Table 6:** Multi-phenotype SNPs and multi-phenotype loci information for 21 chlorophyll fluorescence phenotypes

Multi Phenotype SNP	Marker	Site	Chromosome	Multi Phenotype locus	# Traits	Phenotypes
1	ss715578706	2,194,371	1	1	3	F_J_, F_0_, M_0_
2	ss715582243	4,188,852	2	2	3	F_I_, F_J_, F_M_
3	ss715588144	45,019,738	4	3	3	F_V_/F_M_, φ_Do_, φ_Po_
4	ss715598351	5,523,614	7	4	4	M_0_, φ_Eo_, ψ_0_, V_J_
5	ss715602131	43,896,715	8	5	4	φ_Eo_, PI_ABS_, ψ_0_, V_J_
6	ss715606550	37,824,499	10	6	5	ABS/RC, DI_0_/RC, M_0_, φ_Eo_, V_J_
7	ss715611132	7,673,775	11	7	5	ABS/RC, DI_0_/RC, F_V_/F_M_, φ_Do_, φ_Po_
8	ss715611140	7,753,142			7	ABS/RC, DI_0_/RC, F_M_/F_0_, F_V_/F_M_, F_V_/F_0_, φ_Do_, φ_Po_
9	ss715611141	7,763,083			5	ABS/RC, DI_0_/RC, F_V_/F_M_, φ_Do_, φ_Po_
10	ss715611188	8,100,166			7	DI_0_/RC, F_M_/F_0_, F_V_/F_M_, F_V_/F_0_, φ_Do_, φ_Po_, PI_ABS_
11	ss715611189	8,100,210			7	DI_0_/RC, F_M_/F_0_, F_V_/F_M_, F_V_/F_0_, φ_Do_, φ_Po_, PI_ABS_
12	ss715613004	3,977,509	12	8	6	F_M_/F_0_, F_V_/F_0_, M_0_, φ_Eo_, ψ_0_, V_J_
13	ss715613007	3,981,255			7	ET_0_/RC, F_M_/F_0_, F_V_/F_0_, M_0_, φ_Eo_, ψ_0_, V_J_
14	ss715613098	4,570,804			5	ET_0_/RC, M_0_, φ_Eo_, ψ_0_, V_J_
15	ss715613099	4,571,455			5	ET_0_/RC, M_0_, φ_Eo_, ψ_0_, V_J_
16	ss715613101	4,592,714			3	ET_0_/RC, ψ_0_, V_J_
17	ss715613103	4,599,923			3	M_0_, ψ_0_, V_J_
18	ss715615442	33,647,377	13	9	3	F_I_, F_M_, TR_0_/RC
19	ss715616214	40,597,986		10	6	ET_0_/RC, F_M_/F_0_, F_V_/F_0_, φ_Eo_, ψ_0_, V_J_
20	ss715622432	49,050,145	15	11	3	ET_0_/RC, ψ_0_, V_J_
21	ss715627511	38,693,686	17	12	3	F_I_, F_J_, TR_0_/RC
22	ss715635516	45,602,382	19	13	3	F_I_, F_M_, F_V_
23	ss715635520	45,643,073			3	F_I_, F_M_, F_V_
24	ss715635524	45,670,756			3	F_M_/F_0_, F_V_, F_V_/F_0_
25	ss715635529	45,771,542			9	ET_0_/RC, F_I_, F_M_, F_M_/F_0_, F_V_, F_V_/F_M_, F_V_/F_0_, φ_Do_, φ_Po_
26	ss715635531	45,785,358			9	ET_0_/RC, F_I_, F_M_, F_M_/F_0_, F_V_, F_V_/F_M_, F_V_/F_0_, φ_Do_, φ_Po_
27	ss715635532	45,790,916			8	F_I_, F_M_, F_M_/F_0_, F_V_, F_V_/F_M_, F_V_/F_0_, φ_Do_, φ_Po_

For presentation purposes, the 21 fluorescence phenotypes examined here were grouped into four categories: Primary fluorescence phenotypes, Relative phenotypes, Extracted phenotypes, and Energy flux phenotypes (Table 2). The MPL associated with nine phenotypes, MPL-13 was located on chromosome 19 and was marked by six SNPs within 0.147 Mb. The nine phenotypes included representatives from all four fluorescence phenotype categories defined in Table 2. All of the Relative phenotypes were associated with MPL-13 and three of the primary phenotypes, F_M_, F_V_ and F_I_. Additionally, two phenotypes from the Extracted phenotypes category were associated with MPL-13, φ_Po_ and φ_Do_, and lastly one phenotype from the Energy flux phenotypes category, ET_0_/RC.

**Fig. 5:**
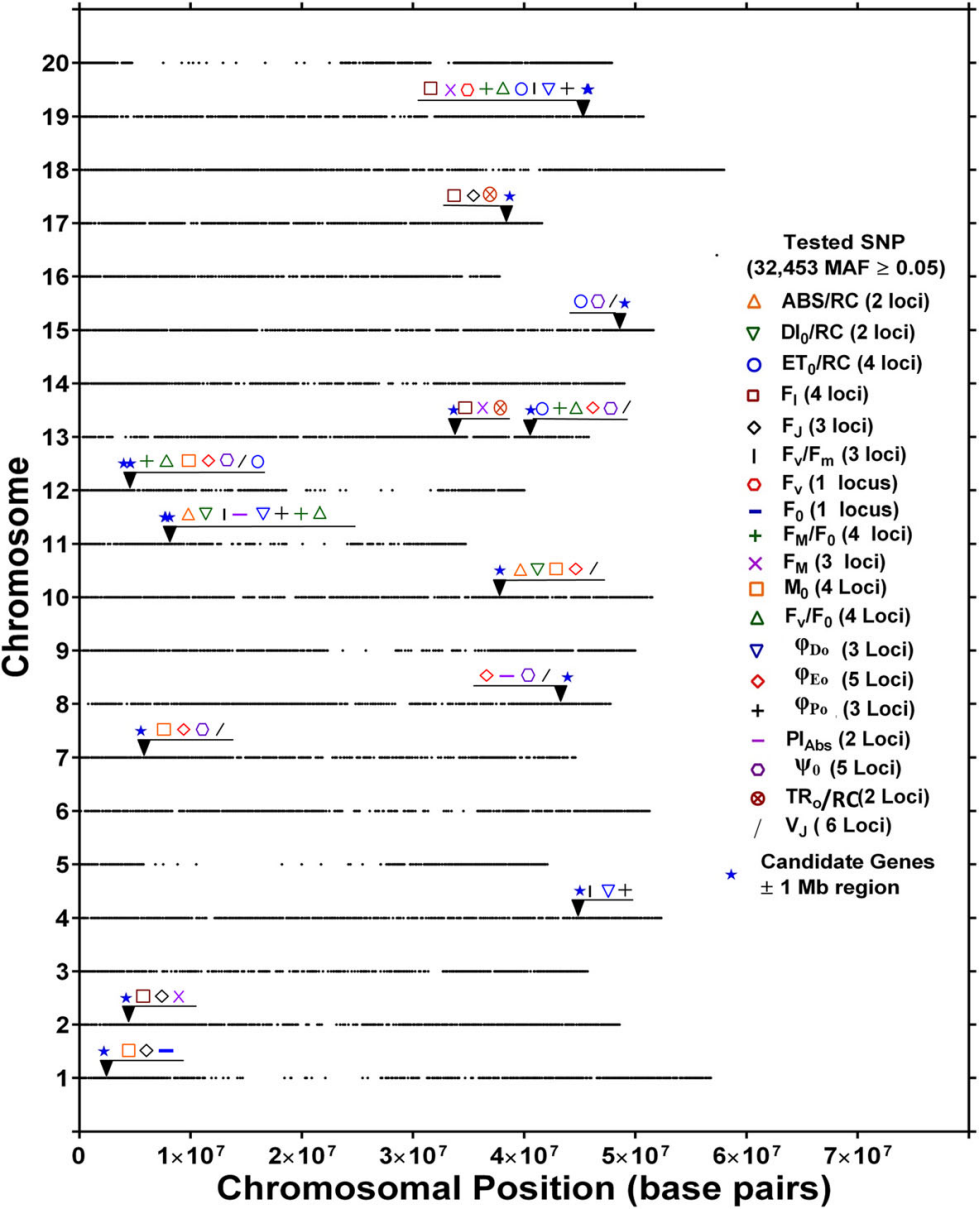
Location of putative loci significantly associated with same SNP identified for at least three phenotypes or more for 19 of 21 fluorescence phenotypes and 30 chlorophyll fluorescence related genes identified in Soybase. Different symbols or colors were used to represent loci identified by respective phenotypes positioned above the respective chromosomes. For each chromosome, the black dots represent the locations of SNPs evaluated for association with chlorophyll fluorescence

The phenotypes F_0_, F_J_, F_I_, F_M_, F_V_, V_J_, and V_I_ were classified as Primary fluorescence phenotypes as they are either the raw fluorescence values at specific times during the fluorescence induction curve or represent variable fluorescence at specific times during the induction curve. All of these phenotypes are influenced by how efficiently the photosystems are with absorbed light energy and the changes in energy [66]. One or more of these traits were associated with 10 of the 13 MPL identified in this study, and, except for V_I_, all of them were associated with at least one MPL (Fig. 5). Variable fluorescence at the J-step was associated with 6 MPL, the largest number of MPL associations among the primary fluorescence traits. Both MPL-2 and MPL-13 were associated with three primary chlorophyll fluorescence traits, including F_I_, F_M_, and F_J_ in the case of MPL-2 and F_I_, F_M_, and F_V_ in the case of MPL-13. Strasser et al. [67] showed that the rise in chlorophyll fluorescence from the J-step to the I-step is associated with the reduction of the plastoquinone pool, and that F_M_ is associated with the reduction of the PSI acceptor side. Accordingly, a gene or genes underlying the association of F_J_ with a locus such as MPL-2 may modulate the redox status of the plastoquinone pool, while MPL-13 or other MPL associated with similar phenotypes could mark genes that influence the PSI acceptor side. The presence of F_I_ in both MPL-2 and MPL-13 together with F_J_ in the case of MPL-2 and F_M_ in the case of MPL-13 indicates that these markers may be useful to influence the redox status and/or pool size of plastoquinone, and thus, light dependent reactions.

Seven chlorophyll fluorescence phenotypes were grouped into the category of Extracted phenotypes, which was the category represented most often across the 13 MPL. Phenotypes encompassed by this category were found 22 times across the 13 MPL and associated with 11 of the 13 MPL. Two of the phenotypes from this category, φ_Eo_ and Ψ_0_, were each associated with five MPL, four of which they had in common. Ψ_0_ is associated with how likely a trapped exciton moves passed the primary electron acceptor of PS2 and φ_Eo_ is associated with the quantum yield of electron transfer. As such, downstream improvement in later stages of electron transport should enhance the likelihood that an electron is passed on from the primary electron acceptor of PS2. Previously, Misra et al. [68] observed that increased salt concentration reduced Ψ_0_ as well as φ_Eo_. Thus, the MPL associated with Ψ _0_ and φ_Eo_ could represent crucial genetic information to improve electron transport efficiencies.

On chromosome 11, MPL-7 was identified by five SNPs within 0.426 Mb and was associated with Relative, Energy flux and Extracted phenotypes (Table 6). The Extracted phenotypes, φ_Do_ and φ_Po_, were associated with all of the SNPs that comprised MPL-7. While the mechanisms underlying NPQ are well understood; however, despite the possible benefits of optimized NPQ for crop production, the identification of useful phenotypes in field grown crops has proven difficult [69]. The association of MPL-7 with φ_Do_, the quantum yield at time 0 for energy dissipation, may be useful to identify genotypes with valuable NPQ phenotypes for use in breeding.

The Relative phenotype category encompasses some of the most commonly reported chlorophyll fluorescence phenotypes. Together, these phenotypes were associated with five of the 13 MPL identified in this study, including 3 for F_V_/F_M_, 4 for F_M_/F_0_, and 4 for F_V_/F_0_ (Fig 5 and Table 6). All three of these phenotypes were associated with MPL-7 on chromosome 11, and Fv/Fm, maximum quantum yield, was associated with four of the five SNPs of this MPL. Of all the chlorophyll fluorescence phenotypes, F_V_/F_M_ is probably the one that has been examined for the most environmental stress factors and across the largest number of species. Many studies examining plant responses to abiotic stress report the effects of the stress on F_V_/F_M_, including studies about plant responses to drought stress [59, 70, 71], nutrient availability [27, 72, 73], heat stress [74–76], and salinity [77, 78]. The importance of F_V_/F_M_ as an indicator of photochemical efficiency and the often-observed impact of environmental stress conditions on F_V_/F_M_ draw attention to the potential utility of identifying loci associated with this phenotype as they may prove valuable to optimize photochemical efficiency in soybean germplasm. Previously, QTL for F_V_/F_M_ were reported for soybean by Yin et al. [30] and by Hao et al. [32], and by Yang et al. [54] in wheat. Yin et al. [31], found one F_v_/F_M_ QTL on chromosome 7 in soybean, and Hao et al. [32] reported an individual SNP associated with F_V_/F_M_ on chromosome 4, but neither were located in the proximity of MPL or other loci associated with F_V_/F_M_ in the present study. As such, the F_V_/F_M_ loci identified here are novel and different from those reported by Yin et al. [31] and Hao et al. [32]. In part, this lack in overlap among loci may be due to genotype by environment interactions and/or differences in timing of phenotyping between the present study and those of Yin et al. [31] and Hao et al. [32] who phenotyped at full seed when net photosynthetic rates and source-sink relationships are considerably different [79].

Energy absorption per reaction center by antenna (ABS/RC), TR_0_/RC, ET_0_/RC, and DI_0_/RC were grouped as energy flux phenotypes and as a group were associated with, eight of the 13 MPL identified. Depending on the energy flux phenotype associated with these MPL, the genes underlying these associations could be important for improving energy absorption, energy trapping, and energy flux for electron transport or the ratio of dissipated energy. The energy flux for electron transport per reaction center, ET_0_/RC, was associated with four MPL, and the other three phenotypes with 2 MPL each. The majority of studies that report ET_0_/RC have focused on the effects of different stress factors and found inconsistent responses of ET_0_/RC [80–82] for instance, Mathur et al., observed that heat stress of wheat lead to increases of ET_0_/RC that were due to the reduced number of active reaction centers [83]. Little is known about genotypic variation in ET_0_/RC and the influences observed by Mathur et al. [83] indicate that the turnover of damaged reaction centers could be involved.

Since the phenotypes examined in this study are all describing aspects related to the photosynthetic electron transport chain, the identification of a considerable number of MPL was not surprising. To date, for many of the individual chlorophyll fluorescence phenotypes assessed in this study, the impact of different values on biomass accumulation or grain yield and even photosynthetic rates are not well understood. As such, the 19 loci identified to be associated with individual chlorophyll fluorescence phenotypes should be useful for future efforts aimed at establishing the importance of specific traits relative to plant performance. In addition to loci that could be targeted for in depth studies and to improve individual fluorescence phenotypes or different categories of fluorescence phenotypes in advanced germplasm, MPL represent promising targets to impact several aspects of the light-dependent reactions. Among them, MPL-13, which was associated with the largest number of fluorescence phenotypes (9), is of particular interest. Two of the nine fluorescence phenotypes, F_M_/F_0_ and F_V_/F_0_, are among the more commonly reported traits and were associated with four consecutive SNPs in MPL-13. The phenotype Fv/F_0_, maximum primary yield of photochemistry of photosystem II, was less affected by drought in drought tolerant barley genotypes [59] and more sensitive to cold stress in pea plants (*Pisum sativum L.*) than F_V_/F_M_ [84]. Given these and other reports of links between chlorophyll fluorescence phenotypes, such as Fv/F_0_, with tolerance to drought and cold stress, the MPL-13 on chromosome 19 may represent a useful marker to improve photosynthetic efficiency in soybean under abiotic stress conditions.

Previously Hao et al. [32] and Yin et al. [31] found loci that were associated with multiple chlorophyll fluorescence phenotypes in soybean, and some were located near MPL identified in the present study. On chromosome 4, MPL-3 was associated with F_V_/F_M_, φ_Eo_ and φ_Po_ and a locus previously identified by Hao et al. (2012) was located approximately 1.5 Mb from MPL-3 and was associated with ABS/RC and ET_0_/TR_0_. Additionally, Hao et al. [32] reported a locus on chromosome 15 that was associated with ET_0_/ABS, ET_0_/TR_0_ and PI_ABS_, and was within approximately 1.4 Mb of MPL-11 which was associated with ET_0_/RC, Ψ_0_ and V_J_. Although the phenotypes that Hao et al. [32] found to be associated with loci near MPL-3 and MPL-11 were not the same as those reported here, their physiological relevance may be similar. Additionally, the relatively short distance between two loci reported here and by Hao et al. [32], provides additional support for the presence of genes of interest in those regions of chromosomes 4 and 15. One of the QTL reported by Yin et al. [31] was located 2.1 Mb from MPL-6 but was not associated with similar phenotypes. Although, they identified a QTL for F_V_/F_M_ on chromosome seven, it was more than 12 Mb from MPL-4 which was not associated with F_V_/F_M_.

### Candidate genes

Regions of the genome marked by MPL may contain groups of genes that are important for different chlorophyll fluorescence phenotypes, or alternatively, these regions may encompass single genes that have a significant impact on photosynthetic light dependent reactions, and thus, influence the values of multiple chlorophyll fluorescence phenotypes. Examination of gene annotations in the vicinity of the 13 MPL identified in this study revealed 30 candidate genes (Table 7). Of the 30 genes, those with annotations indicating involvement in chlorophyll biosynthesis formed the largest group (Table 7). A study using barley found chlorophyll content to be significantly correlated with F_0_ under well-watered conditions [52]. The panel of 189 genotypes utilized in the present study came from a larger set of soybean genotypes that differed in chlorophyll content by 25.9 μg cm^− 2^ [85]. Given the result of Guo et al. [62] and the considerable variation found by Singh et al. [85] et a, it is likely that chlorophyll content of the genotypes affected chlorophyll fluorescence. Interestingly, one of the candidate gene located near MPL-9 on chromosome 13 (Glyma.13G232500) was annotated as CHLI subunit of magnesium chelatase. Magnesium chelatase is responsible for catalyzing the branch point separating chlorophyll biosynthesis from heme synthesis [86]. Plants with mutations in enzymes of the metabolic pathway for chlorophyll also have altered chloroplast morphology. The gene Glyma.13G215400 located near MPL-9 on chromosome 13 was annotated as AT5G18660. The *Arabidopsis thaliana* mutant of AT5G18660, pale-green and chlorophyll *b* reduced 2, was identified based on a lack of grana and was found to accumulate divinyl chlorophylls [87]. The accumulation of divinyl chlorophylls, at the expense of chlorophyll b, had a profound effect on grana stacking, which is critical for efficient photosynthesis. The numerous candidate genes identified that are thought to be involved with chlorophyll biosynthesis, indicate that complex interactions may contribute to perturbations along the pathway of chlorophyll biosynthesis that impact the photosynthetic efficiencies of different genotypes. These relationships among candidate genes, chlorophyll content, and chlorophyll fluorescence phenotypes will need to be investigated further to identify advantageous alterations in chlorophyll biosynthesis that affect photosynthetic efficiency.

**Table 7:** Putative candidate genes associated with chlorophyll fluorescence phenotypes based on the multi phenotype loci (MPL)

Gene ID	Chromosome	Start	End	Soybase	Mb to nearest MPL-SNP	Functional annotation	Annotation category
Glyma.01G017400	Gm01	1,653,678	1,657,140	Glyma 2.0	0.5407	RNA-binding (RRM/RBD/RNP motifs) family protein	Nucleotide Binding
Glyma.01G028900	Gm01	3,060,271	3,064,492	Glyma 2.0	0.8659	ATP-citrate lyase A-1	Chlorophyll biosynthesis
Glyma.02G036300	Gm02	3,341,760	3,346,084	Glyma 2.0	0.8471	ATP-citrate lyase A-1	Chlorophyll biosynthesis
Glyma.02G047600	Gm02	4,376,285	4,379,912	Glyma 2.0	0.1874	Ferredoxin-NADP(+)-oxidoreductase 1	Biochemical
Glyma.07G057200	Gm07	5,078,005	5,083,913	Glyma 2.0	0.4456	THYLAKOID RHODANESE-LIKE, TROL^a^	Protein stability
Glyma.07G059600	Gm07	5,317,552	5,319,355	Glyma 2.0	0.2061	NdhV	Protein stability
Glyma.10G153100	Gm10	38,772,208	38,774,371	Glyma 2.0	0.9477	Photosystem II reaction center PsbP family protein	PSII protein
Glyma.11G094700	Gm11	7,190,632	7,195,838	Glyma 2.0	0.9096	Hydroxymethylbilane synthase.	Chlorophyll biosynthesis
Glyma.11G100800	Gm11	7,629,148	7,632,965	Glyma 2.0	0.4711	Photosystem II stability/assembly factor, chloroplast (HCF136)	PSII stability
Glyma.11G108800	Gm11	8,294,535	8,297,524	Glyma 2.0	0.1943	IMPAIRED SUCROSE INDUCTION 1^a^	Photosynthetic gene regulation
Glyma.11G108900	Gm11	8,299,154	8,302,660	Glyma 2.0	0.1989	pfkB-like carbohydrate kinase family protein	Photosynthetic gene regulation
Glyma.11G110000	Gm11	8,387,146	8,394,939	Glyma 2.0	0.2869	NAD(P)-binding Rossmann-fold superfamily protein. GIANT CHLOROPLAST 1	Chloroplast structure
Glyma.11G111100	Gm11	8,483,000	8,499,771	Glyma 2.0	0.3828	Fructose-bisphosphate aldolase 2	Calvin cycle
Glyma.11G111400	Gm11	8,496,826	8,499,771	Glyma 2.0	0.3966	Fructose-bisphosphate aldolase 2	Calvin cycle
Glyma.11G112900	Gm11	8,633,078	8,637,349	Glyma 2.0	0.5329	CGLD11, CONSERVED IN THE GREEN LINEAGE AND DIATOMS 11^a^	ATP synthase
Glyma.11G114700	Gm11	8,753,784	8,755,152	Glyma 2.0	0.6536	Photosystem II reaction center protein C	PSII protein
Glyma.11G119300	Gm11	9,067,166	9,070,714	Glyma 2.0	0.9670	CLP protease proteolytic subunit 6	Chloroplast structure
Glyma.12G044500	Gm12	3,230,504	3,235,246	Glyma 2.0	0.7470	CLP protease proteolytic subunit 6	Chloroplast structure
Glyma.12G061600	Gm12	4,499,104	4,499,532	Glyma 2.0	0.1008	Ribulose-bisphosphate carboxylases	Rubisco
Glyma.13G213500	Gm13	32,711,673	32,716,430	Glyma 2.0	0.9357	Deoxyxylulose-5-phosphate synthase	Chlorophyll biosynthesis
Glyma.13G215400	Gm13	32,875,517	32,877,386	Glyma 2.0	0.7719	NAD(P)-binding Rossmann-fold superfamily protein	Chlorophyll biosynthesis
Glyma.13G225000	Gm13	33,791,887	33,794,220	Glyma 2.0	0.1445	Aldolase-type TIM barrel family protein	Chlorophyll biosynthesis
Glyma.13G232500	Gm13	34,356,666	34,359,254	Glyma 2.0	0.7093	CHLI subunit of magnesium chelatase	Chlorophyll biosynthesis
Glyma.13G299200	Gm13	39,750,734	39,751,715	Glyma 2.0	0.8473	Photosystem II Psb27	PSII protein
Glyma.13G302100	Gm13	39,924,081	39,927,914	Glyma 2.0	0.6739	Photosystem II reaction center PSB29 protein	PSII protein
Glyma.13G302900	Gm13	39,981,657	39,984,723	Glyma 2.0	0.6163	CYTOCHROME B-C1 COMPLEX SUBUNIT RIESKE	Cytochrome B6f
Glyma.15G253700	Gm15	48,243,955	48,247,144	Glyma 2.0	0.8062	Photosystem II subunit R	PSII protein
Glyma.15G262700	Gm15	49,552,224	49,552,328	Glyma 2.0	0.5021	Photosystem II reaction center protein T	PSII protein
Glyma.17G228700	Gm17	38,364,416	38,367,780	Glyma 2.0	0.3293	Cytokinin-responsive gata factor 1	Chlorophyll biosynthesis
Glyma.19G212800	Gm19	46,633,685	46,639,818	Glyma 2.0	0.8428	Sucrose synthase 3	Starch synthesis

^a^Indicate annotations refined with the highest scoring homolog from Arabidopsis on www.arabidopsis.org

The second largest group of candidate genes located near MPLs were proteins of the PSII complex (Table 7). Several PSII complex proteins are known to influence chlorophyll fluorescence phenotypes, including PsbR, PsbQ and PsbP [88–90]. PsbP is a protein that participates in the regulation of the oxygen evolving complex and a candidate PsbP encoding gene (Glyma.10G153100) was found near MPL-6 on chromosome 10. Previously, employing an RNAi knock down approach, Yi et al. [89] showed that genotypes with reduced expression of PsbP had reduced F_V_/F_M_ as well as altered chlorophyll fluorescence induction. The PsbP knock down mutants also had altered growth characteristics when not grown on sucrose containing media and was most pronounced with mutants that displayed less PsbP levels. Thus, the loci identified here may be used to identify variations in the PsbP gene that may be useful to improve photosynthetic efficiency. Additionally the PS2 core protein T candidate, PsbT, has previously been shown to be involved with the assembly and efficiency of PS2 [91]. Mutants of PsbT had reduced levels of PS2, oxygen production and altered decay kinetics of fluorescence compared to wild type [91].

Some candidate genes were not directly associated with the light dependent reactions. These candidate genes such as Glyma.12G061600 and Glyma.11G108800 were involved with reactions that utilize the products of the light dependent reactions. The most studied enzyme associated with the light independent reactions is Ribulose-bisphosphate carboxylase (Rubisco) and has been the target of a plethora of investigations on improving photosynthesis [92–94]. The candidate gene, Glyma.12G061600, was located near MPL-8 and was annotated to the large subunit of Rubisco [95], ATCG00490.1. Xu et al. (2009) demonstrated that an inducible glycolate oxidase suppression leads to reductions in F_V_/F_M_, net photosynthesis, Rubisco activase gene expression and Rubisco activity. [95]. The characterization of Impaired Sucrose Induction-1, ISI1, by Rook et al. [96] showed that mutants displayed increased quantum yield and photosynthetic quenching. Genes such ISI1 around MPL-7 could be influencing the sensing of source leaves sugar status and have impacts on chlorophyll fluorescence. Thus, MPL-7 and MPL-8 could be applied to improving the sugar status of leaves to allow for light dependent reactions that are more efficient.

## Conclusion

Chlorophyll fluorescence measurements on 189 MG IV soybean genotypes grown in four different field environments demonstrated high diversity across 21 chlorophyll fluorescence phenotypes. Genome-wide association analysis based on these genotypes and chlorophyll fluorescence phenotypes lead to the identification of 153 unique SNPs. Based on these SNPs, 13 loci associated with at least three phenotypes (multi phenotype loci) were identified. Despite the low heritabilities of Energy flux traits the Primary and Relative fluorescence phenotypes have in some cases much higher values. A search in the genomic regions highlighted by these 13 MPL identified genes with annotations indicating involvement in photosynthetic light dependent reactions. These, as well as loci associated with only one or two chlorophyll fluorescence traits, should be useful to develop a better understanding of the genetic basis of photosynthetic light dependent reactions as a whole as well as of specific components of the electron transport chain in soybean. Since many chlorophyll fluorescence traits are responsive to changes in environmental conditions, careful characterization of the loci identified here may lead to avenues for improved stress tolerance. Accordingly, additional genetic and physiological analyses are necessary to determine the relevance and effectiveness of the identified loci for crop improvement efforts.

## Additional files

Additional file 1: **Table S1.** List of all significant SNP markers for 21 chlorophyll flourescence phenotypes evalauted in the study. (XLSX 43 kb)

Additional file 2: **Table S2.** List of putative loci significantly associated with same SNP identified for at least three phenotypes or more for 19 of 21 fluorescence phenotypes. (XLSX 32 kb)

## Abbreviations

BLUPs: Best linear unbiased predictions
CMLM: Compressed mixed linear model
DAP: Days after planting
GRIN: Germplasm Resources Information Network
GWA: Genome-wide association
GWAS: Genome-wide association study
H^2^: Broad sense heritability estimate
K: Kinship matrix
MAF: Minor allele frequency
MCMC: Markov chane Monte Carlo
MG: Maturity group
MPL: Multi phenotype loci
NPQ: Non-photochemical quenching
PAR: Photosynthetically active radiation
Q: Population structure matrix
Q_A_: Primary quinone electron acceptor
QTL: Quantitative trait loci
ROS: Reactive oxygen species
SNP: Single nucleotide polymorphism
USDA: United States Department of Agriculture
